# Mask-Assisted Electrochemical Micro-Etching with Self-Circulating Flushing Based on a Pulsating-Cycle Cathode

**DOI:** 10.3390/mi17080942

**Published:** 2026-08-07

**Authors:** Haifeng Zhou, Guoqian Wang, Fengkai Liu, Yan Zhang

**Affiliations:** College of Mechanical and Power Engineering, Nanjing University of Technology, Nanjing 211816, China

**Keywords:** pulsating-cycle cathode, coupled electric-fluid simulation, self-circulating electrolyte, microstructure uniformity

## Abstract

The uniformity of microstructural formation remains a critical challenge in mask-assisted electrochemical micro-etching (M-ECME), primarily governed by electric field and flow field distribution. Implementation of a pulsating-cycle cathode in M-ECME enabled self-sustained pulsed machining. Multiphysics simulations verified control over current and flow fields, while theoretical analysis established 0.253 mm as the optimal activation gap. The cyclically moving cathode acts like a piston to refresh the electrolyte in the machining zone, and cathode velocity should be limited to below 0.16 m/s to prevent cavitation in the flow field. Subsequent machining experiments strongly validated these theoretical findings. Optimized parameters (15 V, 0.14 m/s) produce highly consistent microstructures (245.28 ± 5.50 μm diameter, 50.34 ± 5.49 μm depth) with significantly improved dimensional control.

## 1. Introduction

Surface microstructures have gained extensive applications in aerospace, defense equipment, and medical devices owing to their multifunctional properties [1]. Given their minute feature sizes, intricate geometries, and stringent precision requirements, manufacturing techniques are imperative for microstructure fabrication [2,3]. Some methods such as photolithography and laser etching, despite their high precision, suffer from limitations including exorbitant equipment costs, restricted material compatibility and thermal damage [4,5]. Among various machining technologies, electrochemical machining (ECM) has emerged as a promising approach for efficient microstructure fabrication due to its non-contact nature, absence of residual stress, and broad material applicability (e.g., stainless steel and nickel-based alloys) [6,7,8]. However, with the escalating demands of precision manufacturing, ECM faces dual challenges: further enhancing machining accuracy and achieving flexible production capabilities [9,10].

Within the realm of ECM, extensive research has been conducted to improve machining precision, particularly focusing on the interelectrode gap (IEG). Sapre et al. [11] observed in electrochemical grinding that an excessive IEG reduces shear stress, adversely affecting medical device fabrication. D. Wang et al. [12] studied IEG control in counter-rotating ECM (CRECM) for elliptical anode leveling, demonstrating that maintaining a small IEG (0.22 mm) enhances workpiece roundness. Kong et al. [13] introduced a hybrid ECDM-ECM mode to eliminate discharge-induced recast layers. By adopting a sinking-push approach, the traditional issue of IEG enlargement during material removal was mitigated, enabling high material removal rates (MRRs) and superior surface finish in a single process. It is evident that reducing the machining gap in electrochemical machining (ECM) can significantly enhance processing quality. Consequently, numerous researchers have attempted to study extremely small machining gaps (≤0.1 mm).

However, an excessively small gap inevitably deteriorates the electrolyte flow field. Maintaining optimal flow conditions under such constrained gaps remains a pivotal challenge for achieving high-precision electrochemical machining. Zhang et al. [14] designed an innovative cathode tool for electrochemical milling, enabling ultra-narrow-IEG (0.05 mm) operation. This refinement increased MRR by 107%, reduced surface roughness by 86.2%, and minimized stray corrosion by 60.1%. Volgin’s BEM-FMM [15] analysis of eccentric rotating electrodes with partial insulation (θTE = 10°, S_0_ = 0.001–0.1 R) shows fivefold peak current density increases while maintaining 50% average current (KWP = 0.5) through edge compensation. In addition, the moving-coordinate modeling adopted by Volgin’s research team [16] (Smin = 0.03 R) further reduces shape errors to 0.76 times the material removal thickness (Δ = 0.76 L). For 6061 aluminum ECM, Zhang et al. [17] eliminated 45 μm convex flow marks caused by viscous byproducts in low-flow zones by implementing gradual anode transitions (0.5 mm/2 mm slopes). Painuly et al. [18] proposed an integrated approach that achieved a 129% improvement in material removal rate (MRR) and nanoscale surface quality (Ra ≤ 53.6 nm) by combining precision interelectrode gap (IEG) control (±10 μm), electrolyte optimization, and hybrid techniques (vibration, magnetic, laser). This method enabled micron-scale fabrication for aerospace and medical applications. Furthermore, the reciprocating oscillation of the cathode enables a variable machining gap, which—when synchronized with discharge cycles—permits machining at extremely small gaps while maintaining effective flushing with enlarged gaps. Wang et al. [19] developed an ECM system with an observable IEG to investigate the impact of cathode vibration on gap distribution, revealing that vibration reduces the IEG, increases current density, and enhances blade profile integrity and surface quality. Similarly, Wang et al. [20] proposed a variable-parameter ECM strategy for blisk machining, incorporating micro-vibration and short-pulse coupling. By optimizing parameters to minimize IEG, contour accuracy and surface quality improved by over 50% compared to conventional methods. Existing studies confirm that the reciprocating cathode mechanism effectively reconciles the conflicting requirements of minimal interelectrode gaps and maintained flow field integrity. In addition to mechanical motion-based methods, novel electrode designs have also been explored to enhance ECM precision. For instance, Zhu et al. [21] developed a shunt-assisted silicon electrode that employs an auxiliary anode to mitigate stray current corrosion, demonstrating the ongoing efforts to refine the electrochemical environment for better accuracy.

Nevertheless, achieving flexible manufacturing capability remains a critical obstacle for industrial implementation. The advancement of flexible manufacturing—capable of rapid adaptation to design and production changes—represents a key trend in future industrial development. In ECM, mask-assisted electrochemical micro-etching (M-ECME) exhibits exceptional flexibility for fabricating diverse surface microstructures. For planar processing, mask-based techniques are well-established. Meng et al. [22] achieved micro-pit arrays with a diameter error of 2.49% via M-ECME. Wu et al. [23] fabricated microcell arrays with an average width of 110 μm, depth-to-width ratio of 0.21, aspect ratio of 9000, and dimensional error below 5 μm. Wang et al. [24] proposed a hydrodynamic pressure-assisted scanning cathode method, producing micro-pit arrays with diameters of 385.7 μm and 288.8 μm, depths of 111.8 μm and 40.3 μm, and an etching factor of 1.69. Ke et al. [25] introduced moving-cathode M-ECME, improving uniformity by 68.3% over conventional methods. For 3D structures, Liu et al. [26] developed a two-stage M-ECME process for conical microarrays, achieving structures with a 198 μm height and 28 μm tip diameter. However, these impressive research outcomes still rely on external electrolyte circulation systems, thereby inevitably increasing both process preparation time and costs.

Studies reveal that precise control of the IEG is paramount for dimensional accuracy in M-ECME, while simplification of auxiliary process equipment contributes to improved machining flexibility. Extending the M-ECME methodology, we propose a breakthrough self-circulating M-ECME method based on a pulsating-cycle cathode. Compared to traditional ECM systems, this method offers three key advantages: (1) ultra-small-IEG operation for higher precision; (2) elimination of external electrolyte circulation—mechanical self-circulation replaces pumps, reducing cost and improving flexibility; (3) compatibility with varied workpiece geometries without fixture modification. This mechatronic–hydraulic integrated design establishes a new paradigm for precision ECM.

## 2. Principle and Physical Model of Self-Circulating M-ECME

### 2.1. The Self-Circulating M-ECME Principle

**Figure 1:**
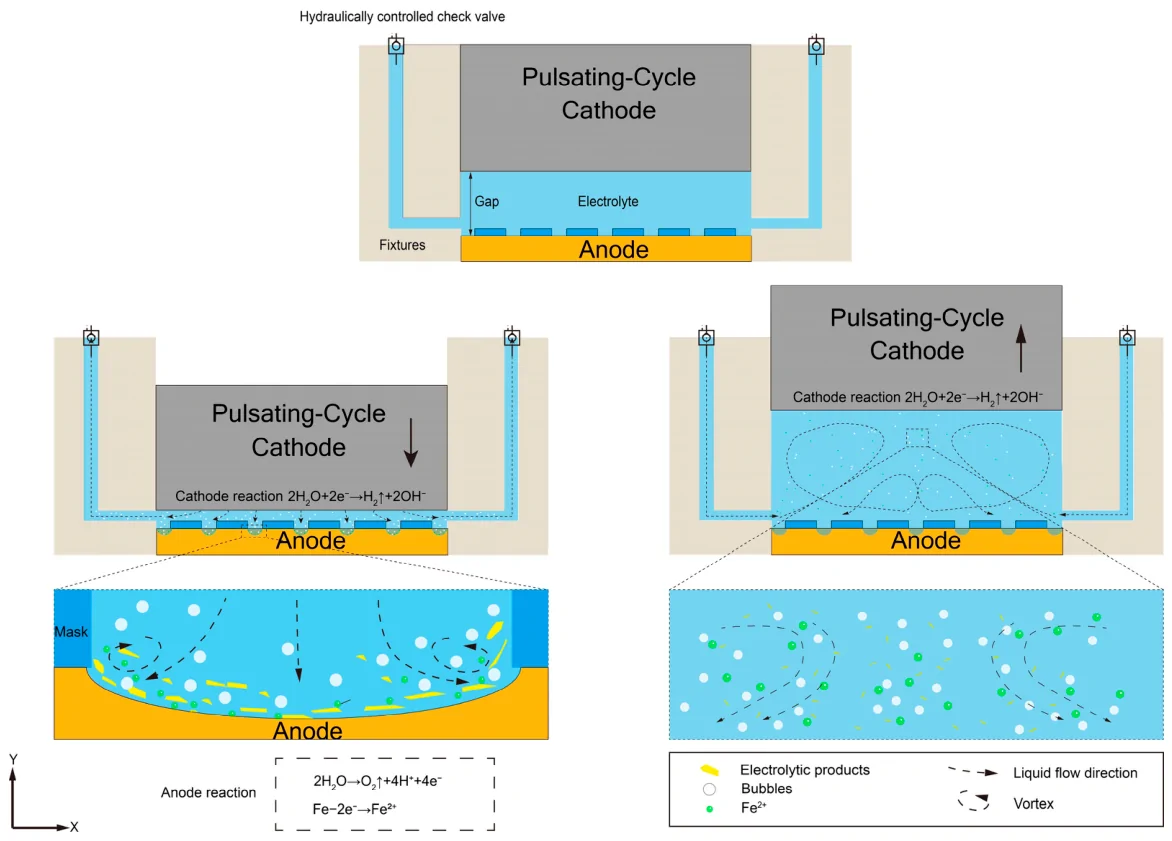
Schematic of self-circulating M-ECME.

The fundamental principle of self-circulating flushing M-ECME is based on achieving dynamic pulsating-cycle modulation of the interelectrode gap through cathode reciprocating motion. This reciprocation simultaneously generates pressure differentials that drive autonomous self-circulating electrolyte circulation within the processing zone. As illustrated in Figure 1, the pulsating-cycle gap variation confines the electrochemical reactions within a precisely controlled narrow gap range. The self-circulating electrolyte circulation eliminates dependence on external pumping systems while maintaining effective electrolyte renewal, thereby significantly simplifying the overall processing system architecture.

### 2.2. Establishing the Coupled Electric–Flow Field

To systematically investigate the influence of the cathode–workpiece gap on the coupled electric–flow field distribution in self-circulating pulsed electrochemical machining, this study conducted comprehensive numerical simulations using COMSOL Multiphysics software (version 6.3). To balance computational accuracy with efficiency, we propose the following plausible assumptions:

(1) The surfaces of the electrodes are defined as equipotential with different electric potentials;

(2) The conductivity k of the electrolyte is constant;

(3) The distribution of current density obeys Ohm’s law.

It should be noted that the assumption of constant conductivity is a simplification that may affect the accuracy of current density prediction under extreme conditions, but its influence is considered acceptable within the scope of this comparative study.

**Figure 2:**
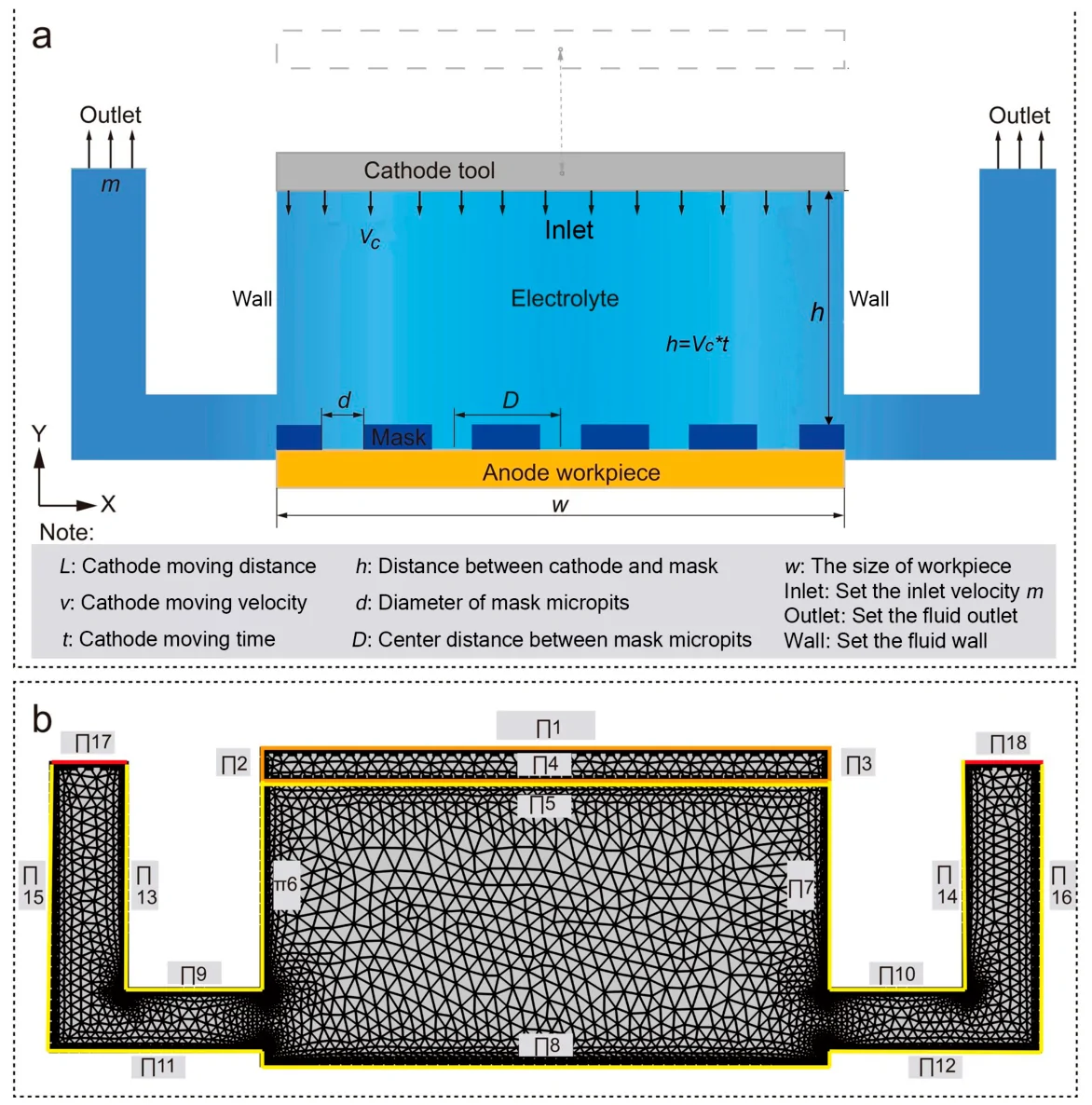
Multiphysics simulations model: (**a**) Electric field model. (**b**) Analog boundary setting.

Based on machining principles and assumed conditions, we selected a representative cross-section of the actual machining region for multiphysics modeling (Figure 2). The electrostatic potential distribution in the interelectrode domain is governed by Laplace’s equation:

(1) $$\frac{\partial^{2}\varphi }{\partial x^{2}}+\frac{\partial^{2}\varphi }{\partial y^{2}}=0$$

The simulation model configuration (Figure 2a) illustrates the cathode tool performing reciprocating motion along the *Y*-axis with an adjustable amplitude h (the distance between cathode and mask: 0–40 mm) and velocity vc. Key geometric parameters included (microhole diameter) and D (hole spacing). The electrical potential conditions are defined as follows:

(2) $$\mathrm{Anode workpiece}:\varphi |\Pi_{8}=U_{a}$$

(3) $$\mathrm{Cathode}\;\mathrm{tool}:\;\varphi |\Pi_{1-4}=0$$

(4) $$\mathrm{Cavity}\;\mathrm{boundary}:\;\frac{\partial \varphi }{\partial_{e}^{\to }}|\Pi_{5-7,9-18}=0$$

(5) $$\mathrm{Shift}\;\mathrm{speed}\;\mathrm{of}\;\mathrm{conductive}\;\mathrm{cathode}:\;\partial v|\Pi_{1-4}=v_{c}$$

where $U_{\;a}$ is the voltage applied to the anode workpiece, and $\vec{e}$ is the unit vector. $v_{c}$ is converted by the motor driving the cathode.

The formula for surface current density is as follows:

(6) $$i_{e}^{\to }=k\nabla \varphi_{e}^{\to }=kE$$

For the flow field in the machining region, the electrolyte is simplified as an ideal incompressible fluid. Consequently, its flow is governed by the Navier–Stokes equations.

(7) $$\frac{\partial v_{c_{i}}}{\partial x_{i}}=0,\;\frac{\partial v_{c_{i}}}{\partial t}+\frac{\partial (v_{c_{i}}v_{c_{j}})}{\partial x_{j}}=f_{i}-\frac{1}{\rho }\frac{\partial p}{\partial x_{i}}+u\frac{\partial^{2}v_{c_{i}}}{\partial x_{j}\partial x_{j}}$$

where ρ is the electrolyte density, u is the kinematic viscosity coefficient of the electrolyte, $v_{c_{i}}$ is the electrolyte velocity vector, p is the electrolyte pressure, $x_{i}$ is the ith spatial coordinate, and $f_{i}$ is the mass force strength of the fluid (in this paper, gravity is the only mass force acting on the electrolyte, so $f_{i}$ = $g_{i}$).

The cathode tool surface was designated as the flow field velocity inlet (velocity *v*_c_). After specifying the electrolyte composition, structured mesh generation was employed (Figure 2b), incorporating coupled current distribution and moving mesh physics interfaces. The boundary conditions were configured as follows: dynamic mesh surface Π1 was constrained with *Y*-direction velocity (−*v*_c_); surfaces Π2 and Π3 were restricted to zero *X*-direction velocity.

A fluid–structure interaction (FSI) approach was employed, with the cathode domain as a rigid, non-deformable body moving at velocity *v*_c_, while the remaining boundaries were treated as deformable domains. Π5 was set as the flow inlet; Π17 and Π18 were designated as flow outlets. All solid boundaries, including the cathode tool surface and the outer walls of the outlet regions, were assigned no-slip wall conditions. This setup ensured accurate simulation of the coupled fluid dynamics and structural interactions in the electrochemical machining process.

## 3. Multiphysics Coupling Simulation Results

To study the machining behavior in the pulsating-cycle cathode region under multiphysics effects, we analyzed the multiphysics simulation results. The operating parameters were determined as follows: the anode voltage range (9 V–18 V) was selected based on the polarization and current efficiency curves of the stainless-steel material, while the cathode velocity (0.10–0.16 m/s) was designed to avoid cavitation in the piston-like impinging flow field. A comprehensive analysis was then performed to evaluate the impact of the pulsating cyclic cathode on the multiphysics electric and flow fields.

### 3.1. Analysis of Electric Field Simulation Results

**Figure 3:**
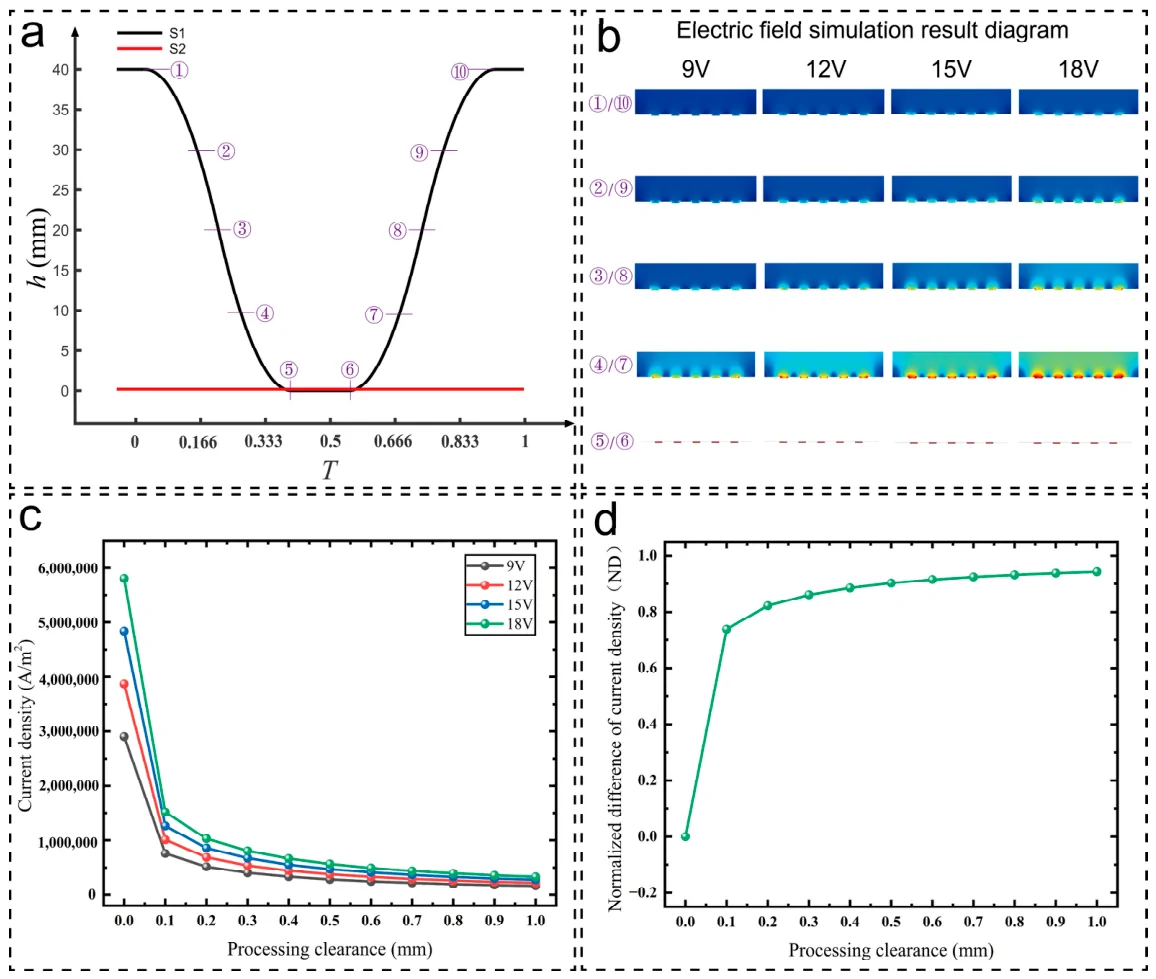
Electric field simulation results: (**a**) Cathode motion trajectory; (**b**) electric field distribution contour; (**c**) variation curve of anode surface current density with cathode motion; (**d**) variation curve of normalized current density difference with cathode motion.

When the pulsating cyclic cathode undergoes periodic motion according to the kinematic curve shown in Figure 3a, the evolution of the electric field distribution within the machining zone is depicted in Figure 3b. Evidently, as the cathode gradually approaches the mask, both the electric field intensity and distribution uniformity are enhanced. The variation in the maximum current density on the anode surface for *h* (the distance between cathode and mask) ≤ 1 mm is plotted in Figure 3c. Clearly, the current density decreases rapidly with increasing *h* under different voltages, and this trend remains independent of the applied voltage magnitude. Furthermore, after normalizing the current density on the anode surface, the curve representing the normalized difference in current density versus *h* under various voltages is shown in Figure 3d. The results demonstrate that smaller machining gaps result in higher current density with improved uniformity. When the machining gap is below 0.1 mm, the current density increases dramatically along with a steep rise in uniformity. Increasing current density and optimizing its uniform distribution can significantly enhance system efficiency and processing accuracy. M-ECME enhances process control through periodic current modulation, where the intermittent off-periods enable self-circulating flushing and byproduct removal, effectively mitigating common issues such as reduced machining efficiency and surface roughness. The precision of ECM depends not only on electrical parameters but also on the flow field.

### 3.2. Analysis of Flow Field Simulation Results

**Figure 4:**
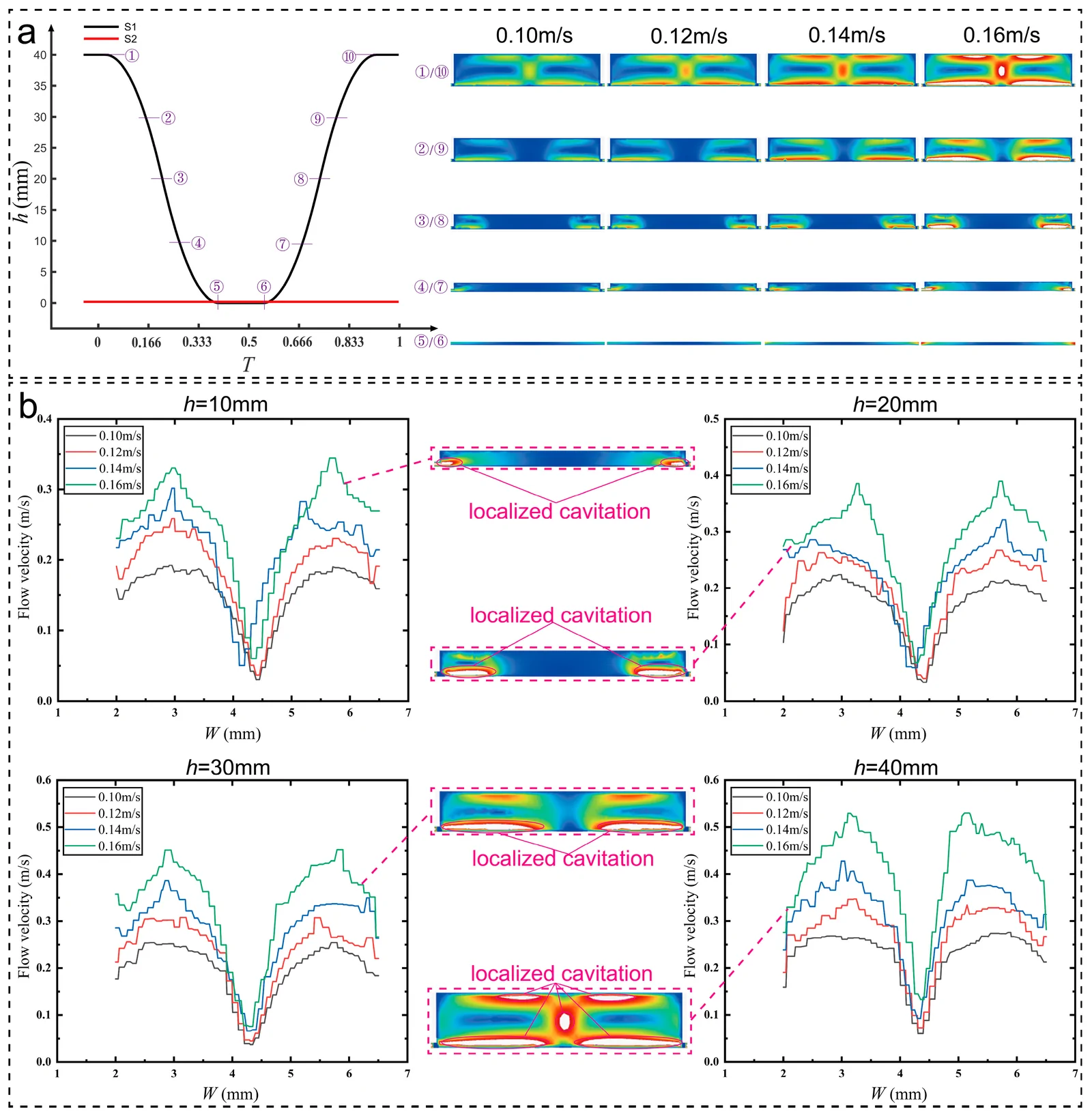
Flow field simulation results: (**a**) flow field distribution contours at different cathode velocities; (**b**) electrolyte flow velocity variation during cathode movement.

While an extremely narrow gap ensures a highly uniform electric field, it simultaneously creates a critical challenge—restricted electrolyte inflow and outflow—which hinders the effective removal of electrolytic byproducts and consequently compromises both machining accuracy and efficiency. To address this issue, we implemented a pulsating-cycle cathode. Its motion pattern is illustrated in Figure 4a, where S1 represents the tool cathode’s movement path and S2 indicates a machining gap of 0.1 mm. The diagram reveals that the optimal machining gap occupies only a small portion of the entire cycle. The majority of the cycle is dedicated to addressing the poor flow field conditions in extremely narrow gaps. During the upward motion, electrolytic byproducts are expelled while fresh electrolyte is replenished to restore ion concentration. This enables more efficient electrochemical reactions during the subsequent downward stroke. Such a cyclic process of machining–flushing–machining effectively mitigates the common issues of byproduct accumulation and inadequate flow field distribution in micro-gap ECM. The simulation results of current field distribution during processing are presented in Figure 4a, where frames 1-5 display the flow field distribution during the cathode’s downward stroke, while frames 6-10 illustrate it during the upward stroke.

The analysis reveals that during the upward motion, higher flow velocities occur at both ends due to fresh electrolyte injection. As the gap increases, the fresh electrolyte gradually converges toward the central region before ejecting, thereby achieving complete electrolyte renewal throughout the entire container. To further investigate how cathode moving speeds affect flow field characteristics across different workpiece surface regions at varying gaps, the experimental results are shown in Figure 4b. The flow field diagrams and velocity curves reveal that increasing the cathode movement speed enhances flow velocity, which rises with increasing gap height *h*. This indicates improved removal efficiency of electrolytic byproducts from micro-cavities. The flow field simulation results confirm that higher cathode travel speeds increase electrolyte flow velocity. However, as is evident from the flow field patterns, the maximum permissible cathode speed is limited to 0.16 m/s due to equipment stability constraints. Consequently, while a cathode rotational speed of 0.16 m/s theoretically yields optimal machining quality, exceeding this speed triggers “localized cavitation effects”. Therefore, selecting a travel speed slightly below and approaching 0.16 m/s proves more advantageous.

### 3.3. Optimization Analysis

Through the above analysis, we can observe that at the optimal gap, there exists the issue of low machining efficiency, while at the optimal machining speed, there is a deterioration in flow field conditions. Therefore, we theoretically investigated their equilibrium—specifically, the relationship among machining speed (workpiece removal rate), effective machining time, and machining gap. The ideal metal removal rate Va decreases to zero as reaction products accumulate. Assuming the anode metal etching speed is c·Va (where c is the removal rate correction factor) during the effective machining time Tf, the removed volume S·n (where S is the removal area and n is the removal thickness) generates k1 times the volume of Fe(OH)_2_ and k2 times the volume of H_2_. If r1 × 100% of Fe(OH)_2_ and r2 × 100% of H_2_ accumulate in the micro-gap, machining becomes unsustainable when exceeding p × 100% of the gap capacity. This establishes the following relationships:

(8) $$S\cdot n\cdot \left(k_{1}\cdot r_{1}+k_{2}\cdot r_{2}\right)=S\cdot ∆\cdot P$$

(9) $$c\cdot V_{a}\cdot T_{f}=n$$

The actual workpiece machining speed is as follows:

(10) $$V_{m}=\frac{n}{T_{f}+T_{0}}=\frac{n}{\frac{n}{c\cdot V_{a}}+T_{0}}=\frac{A\cdot ∆}{\frac{A}{B\cdot c}\cdot {∆}^{2}+T_{0}}$$

In the formula: $A=\frac{P}{k_{1}\cdot r_{1}+k_{2}\cdot r_{2}};B=\eta \omega kR_{R}$, *η* is the current efficiency, *ω* is the volumetric electrochemical equivalent, *k* is the conductivity of the fresh electrolyte, $R_{R}$ is the interpolar ohm drop, ∆ represents the machining clearance, and $T_{0}$ represents the processing fallback time.

The effective machining time is

(11) $$T_{f}=\frac{A\cdot {∆}^{2}}{B\cdot c}$$

**Figure 5:**
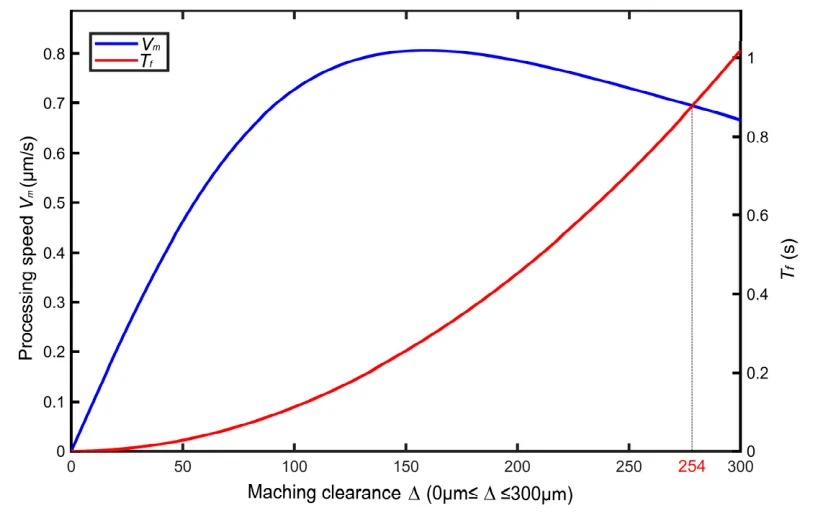
Relationship between processing speed, effective processing time and processing gap.

Through theoretical calculations, Figure 5 was generated, revealing an optimal intersection point between effective machining time and workpiece removal rate at a gap of 253.24 μm.

## 4. Experimental Verification

### 4.1. Experimental Equipment and Processing Procedures

**Figure 6:**
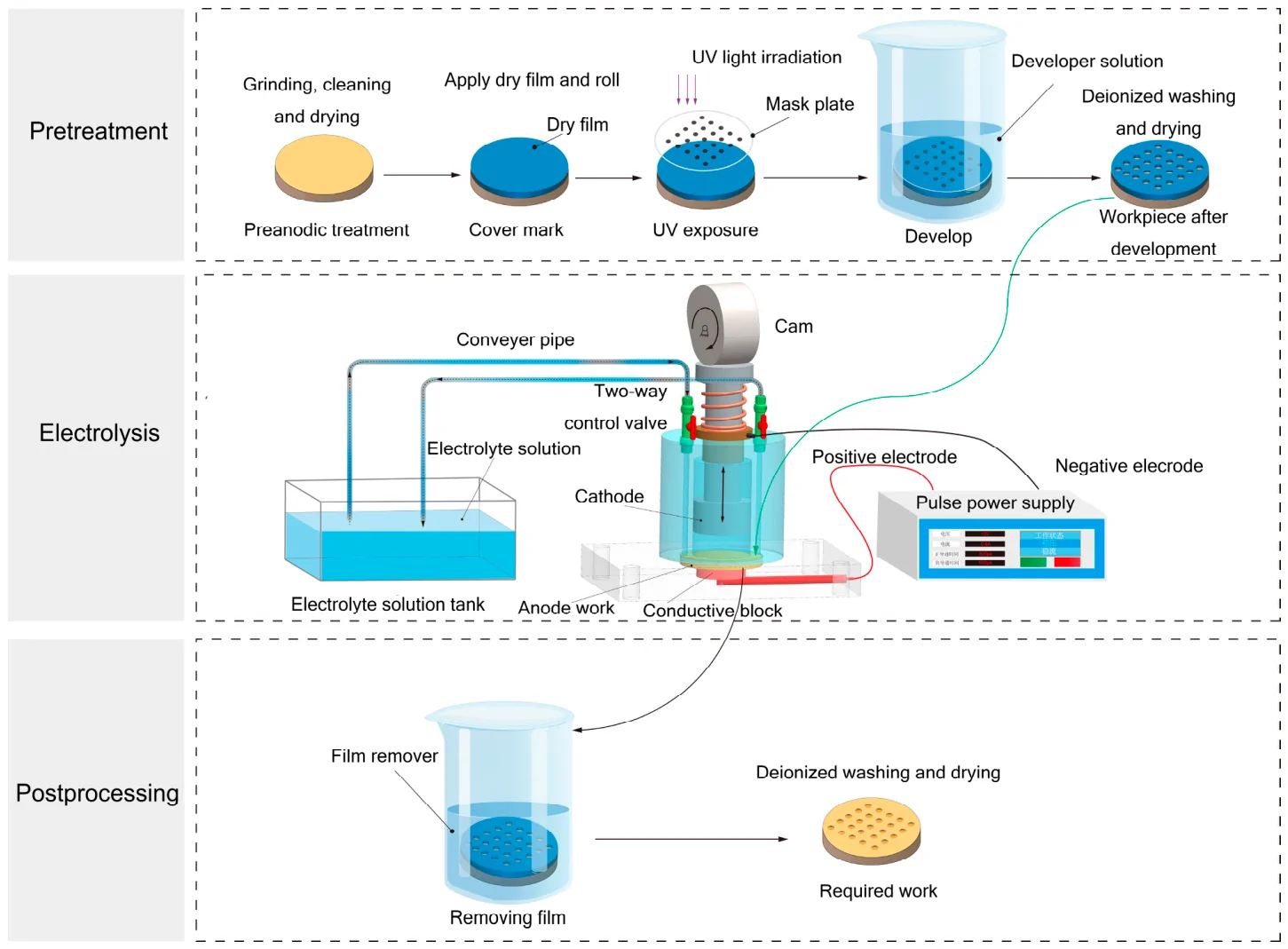
Flow chart of micro-pit arrays manufactured by self-circulating pulsatile mask electrochemical micro-etching technique.

The simulation results indicate that while a reduced gap enhances machining uniformity and the self-circulating M-ECME improves processing efficiency, though experimental validation is needed. Figure 6 illustrates the fabrication process of micro-pit arrays using M-ECME with self-circulating flushing, which consists of three main stages: pretreatment, electrolytic machining, and post-treatment. The pretreatment stage involves dry film lamination, mask photolithography, and development. Specifically, a UV-positive photoresist dry film (DuPont SD230) is used for lamination, followed by mask patterning via a UV exposure system. The electrolytic machining stage features a proprietary self-circulating flushing device, which utilizes a pulsating cyclic (downward–upward) motion to enable the process. The downward stroke initiates electrochemical machining, while the upward stroke refreshes the electrolyte and removes byproducts. In the post-treatment stage, the resist film is stripped, and the workpiece is rinsed with deionized water, completing the micro-pit array fabrication.

### 4.2. Experimental Methods

As evidenced by the simulation results, material removal volume and machining consistency emerge as two pivotal performance metrics. Figure 5 establishes effective machining time—directly proportional to removal volume—as the efficiency driver, while Figure 3c,d reveal how consistency controls removal uniformity, a prerequisite for precision. The removal volume quantifies dissolved material within a fixed 80 s duration, while the machining consistency assesses surface uniformity under identical conditions. Based on the simulation analysis, the experimental work was organized into five primary groups, comprising twenty sub-groups in total. The detailed parameters for all groups are provided in Table 1. The five primary groups were categorized according to the cathode’s traveling speed. Each primary group was then divided into four sub-groups based on the machining voltage. Among these, Group 0 served as the control group. In this group, the cathode was stationary, and the gap distance (*h*) between the cathode and the mask was maintained constant at 1 mm. For the other four groups, the cathode’s motion pattern was consistent with the simulation conditions.

### 4.3. Experimental Results

Following the experiments, all fabricated microstructures were thoroughly characterized using both scanning electron microscopy (SEM, SU8010, Hitachi High-Tech Corporation, Tokyo, Japan) and confocal laser scanning microscopy (CLSM, OLS5000, Olympus Corporation, Tokyo, Japan) to evaluate key parameters including surface morphology, etching depth, and etching diameter. The processing efficiency is characterized by the etching diameter and depth, while consistency is quantified by their standard deviations.

**Figure 7:**
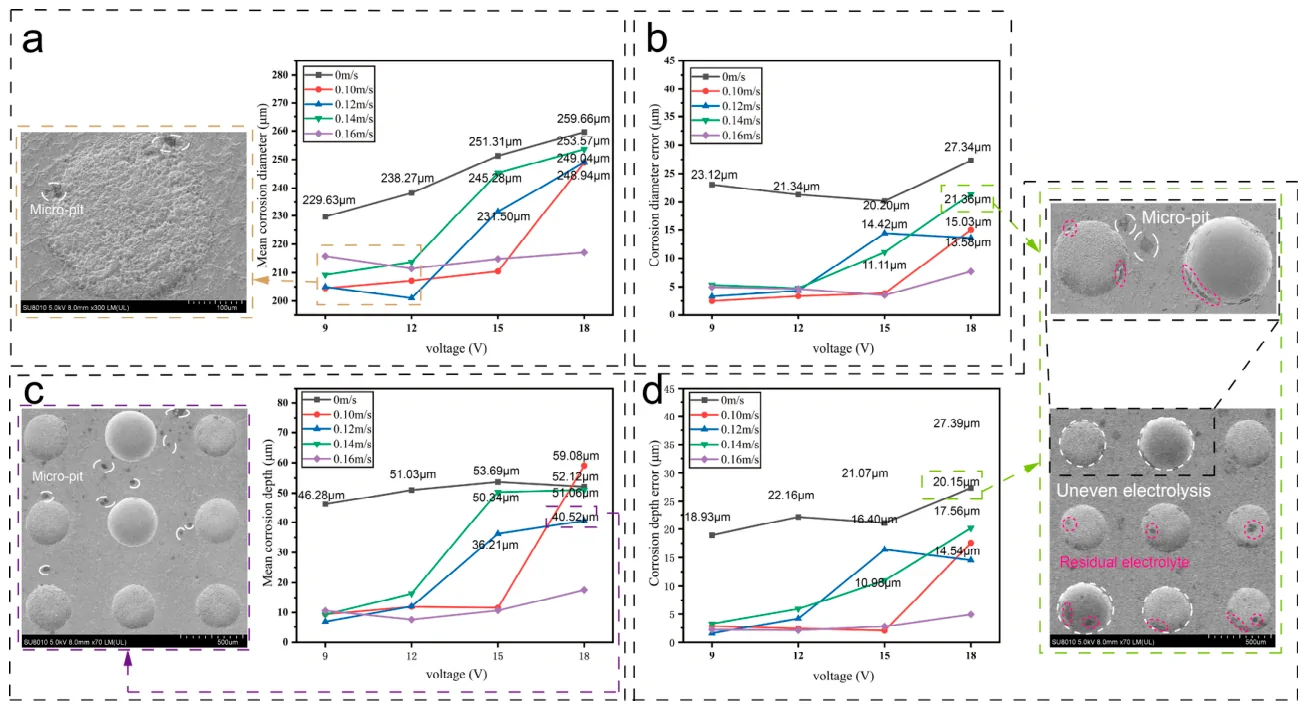
Experimental result diagram: (**a**) Corrosion diameter mean diagram at different cathode moving speeds; (**b**) corrosion diameter deviation diagram at different cathode moving speeds; (**c**) corrosion depth diagram at different cathode moving speeds; (**d**) corrosion depth deviation diagram at different cathode moving speeds.

Figure 7 presents the experimental results: (a) average etching diameter at different cathode travel speeds, (b) standard deviation of etching diameter, (c) average etching depth, and (d) standard deviation of etching depth. When the cathode remains stationary with a fixed interelectrode gap of 1 mm, the machining results exhibit high processing efficiency but insufficient precision. As shown in Figure 7a,c, both the diameter and depth of the micro-dents machined with a stationary cathode are significantly larger than those achieved with a cyclically moving cathode. The underlying reason for this is the continuous material removal with a stationary cathode, whereas the cyclic cathode motion results in a pulsed regime where machining occurs only when the interelectrode gap is minimized. However, comparing the deviations in diameter and depth reveals that machining precision is notably poorer with the stationary cathode. When the cathode is fixed at a 1 mm gap, the edge effect in the electric field distribution becomes prominent. Within the machining zone, the current density is higher at the edges, leading to a faster etching rate and consequently increased machining deviation.

In contrast, when the cathode undergoes pulsating cyclic motion, the machining results differ substantially from those of conventional mask-electrolytic machining, in particular showing a significant improvement in machining precision. As shown in Figure 7a, the average etching diameter increases with voltage across all speed conditions. In the low-voltage range (9–12 V), the diameters remain small (205–215 μm) due to insufficient current density, which results in low electrolytic efficiency. A significant transition occurs at 15 V, where the diameters at 0.12 m/s and 0.14 m/s increase markedly, while the 0.10 m/s case remains small until a sharp rise at 18 V. Simulations indicate that higher cathode speeds enhance flow field intensity, improving byproduct removal and electrolysis efficiency. At 0.16 m/s, the diameter stabilizes around 210 μm—consistent with Figure 4b simulations. Excessive speed induces localized cavitation, impairing electrolyte renewal and reducing etching efficiency. Figure 7b reveals a similar trend: standard deviations stay below 5 μm at low voltages due to shallow etching but gradually increase with voltage, exceeding 10 μm at 18 V for most speeds. This instability likely stems from intensified turbulence or cavitation at higher voltages, disrupting uniform byproduct removal. Meanwhile, Figure 7c demonstrates that etching depth also grows with voltage, particularly surging at 18 V, as the increased electric field strength elevates current density, accelerating material removal. Depth variations among speeds are pronounced, with 0.14 m/s and 0.16 m/s exhibiting greater increases than 0.10 m/s and 0.12 m/s, aligning with the flow velocity trends in Figure 5, where higher speeds enhance electrolyte renewal and etching efficiency. The corresponding standard deviations in Figure 7d remain low at 9–12 V but reach 17.56 μm (0.12 m/s) and 10.98 μm (0.14 m/s) at 15 V.

Overall, etching efficiency remains low below 15 V due to subcritical current density, while beyond this threshold, etching dimensions scale with cathode speed—except at 0.16 m/s, where excessive flow velocity induces localized cavitation effects, limiting effectiveness.

**Figure 8:**
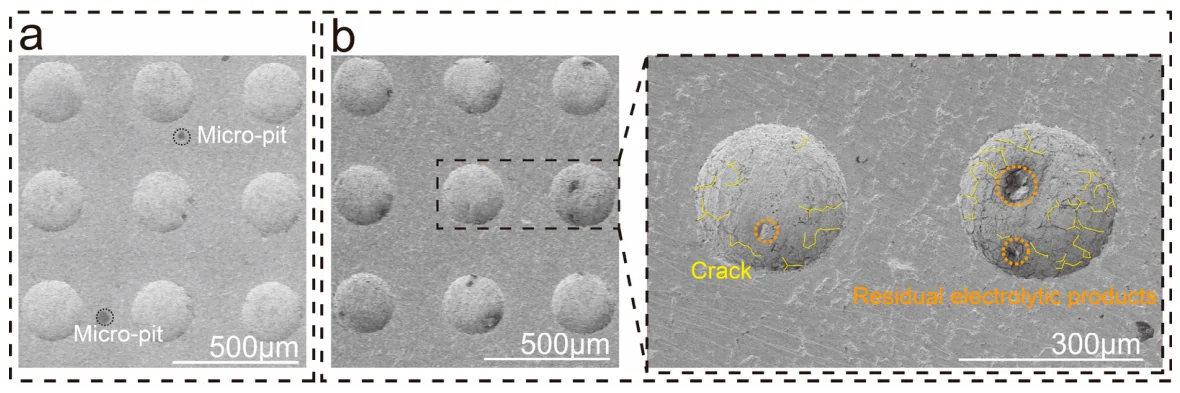
Electrolysis diagrams of the experimental workpieces in Groups 3-3 and 1-4. ((**a**) is the experimental result of Group 3-3 and (**b**) is the experimental result of Group 1-4.)

For parameter optimization, we conducted in-depth analysis focusing on etching depth (Figure 7c). After excluding the inefficient 9–12 V range, comparison between 15 V and 18 V parameters reveals that the 0.16 m/s condition is eliminated due to shallow depth. At 15 V, 0.14 m/s yields better depth and lower standard deviation (10.98 μm) than 0.12 m/s (17.56 μm), making Group 3-3 (15 V/0.14 m/s) a prime candidate. Although the 0.10 m/s condition at 18 V achieves maximum depth, its standard deviation is 7 μm higher than in Group 3-3, necessitating further comparison between these two parameter sets. To systematically evaluate the optimal parameters, we considered four criteria: etching depth, diameter uniformity, array consistency, and surface quality. After excluding the inefficient low-voltage (9–12 V) conditions and the 0.16 m/s group (which induced cavitation), Group 3-3 (15 V, 0.14 m/s) and Group 1-4 (18 V, 0.10 m/s) emerged as the two primary candidates. As shown in Figure 8, although Group 1-4 achieved greater depth, it exhibited poorer circularity and more irregular boundaries, attributed to insufficient flow field intensity at the lower cathode speed. In contrast, Group 3-3 demonstrated superior overall uniformity and better surface quality with slightly shallower depth. Based on this balanced consideration, 15 V and 0.14 m/s are determined as the optimal parameters, consistent with the flow field simulation results. These results clearly demonstrate that the pulsating-cycle cathode travel speed significantly influences the flow field characteristics, which in turn directly affect both the consistency and precision of the M-ECME.

**Figure 9:**
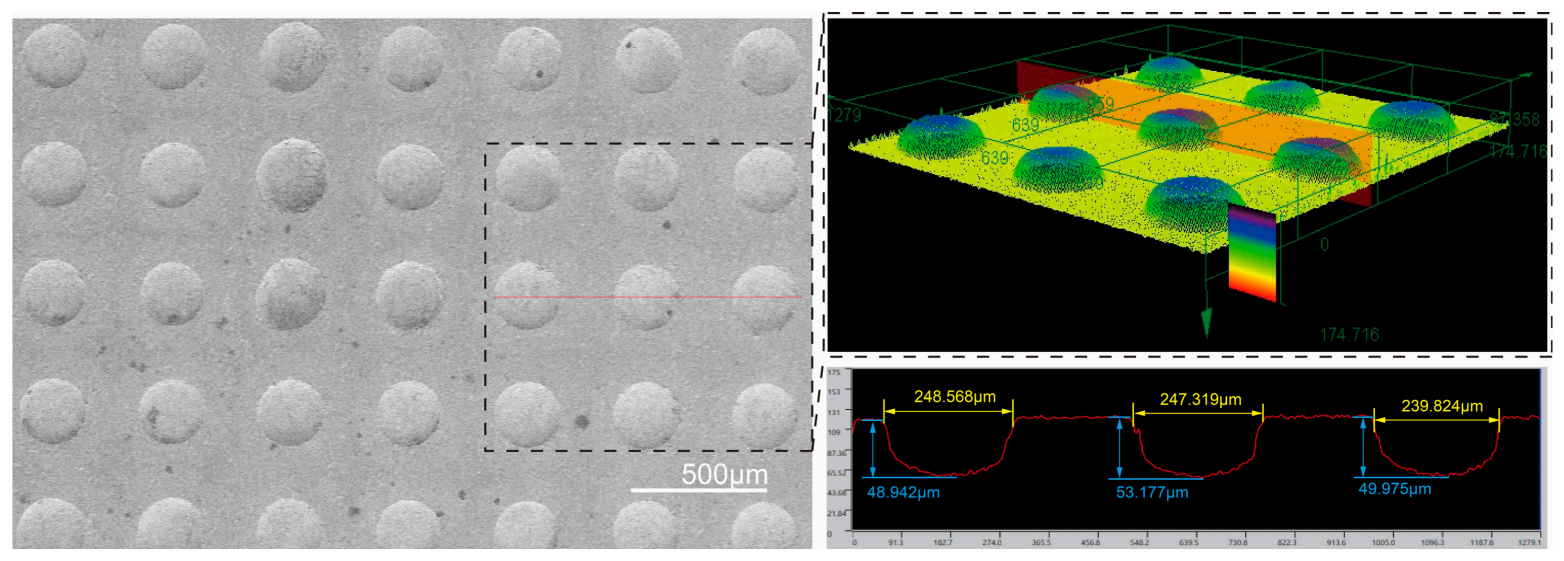
The best-parameter electrolysis result and three-dimensional morphology diagram.

The M-ECME results obtained under optimal parameters and their corresponding three-dimensional topographical characterization are presented in Figure 9. The figure presents SEM images of the array micro-dents and 3D surface topography of selected regions processed with the determined optimal parameters. Experimental results demonstrate that under this parameter combination, the micro-dents exhibit an average diameter of 245.283 μm with a standard deviation of 11.10 μm (<15 μm), and an average depth of 50.344 μm with a standard deviation of 10.97 μm (<15 μm), indicating excellent dimensional consistency in the machined features.

Measurement uncertainties and process variations also contribute to the observed deviations. Systematic errors mainly arise from mask patterning accuracy, cathode–mask alignment offset, and power supply voltage ripple. Random errors include electrolyte temperature fluctuations, stochastic bubble detachment, and CLSM measurement uncertainty. Despite these factors, the optimal parameter set (Group 3-3) consistently outperforms other groups, and the standard deviations remain within acceptable limits for micro-ECM applications.

## 5. Conclusions

This study developed a self-circulating flushing pulsed dynamic machining process based on M-ECME technology to enhance etching accuracy and processing efficiency. The proposed technology utilizes a pulsating cyclic cathode to modulate the machining gap, enabling electrical activation when an extremely small interelectrode gap is achieved. This results in machining with exceptionally high and uniform current density. Simultaneously, the cyclically moving cathode functions as a piston to circulate the electrolyte internally, thereby eliminating the need for external pumping systems and reducing the overall application cost of this technique. Through multiphysics simulation and experimental investigations, the following key conclusions were obtained:

(1) Theoretical analysis suggests that maintaining a machining gap below 0.1 mm can achieve the highest and most uniform current density. However, this extremely narrow gap severely restricts electrolyte renewal and reduces the effective machining time due to rapid byproduct accumulation. By comprehensively considering both current density and effective processing time, we theoretically determined that a gap of 0.253 mm provides the optimal overall performance as a trade-off between electric field uniformity and flushing efficiency. Furthermore, the cathode travel speed shows a positive correlation with flow field velocity. It should be noted that when the travel speed exceeds 0.16 m/s, localized cavitation effects occur, leading to insufficient material removal and consequent degradation of machining quality.

(2) Validation experiments demonstrate that the pulsating cyclic cathode can achieve electrolyte circulation, with faster cathode movement speeds resulting in higher electrolyte flow velocities. However, when the cathode speed exceeds a certain threshold, both machining efficiency and quality deteriorate significantly. This finding aligns with simulation results, indicating that excessive cathode velocity induces localized electrolyte cavitation. Furthermore, the strategy of initiating machining only after the cathode reaches a working gap of 0.253 mm effectively reduces the standard deviation of microstructure dimensions. This confirms the rationality of the effective machining gap determined through theoretical analysis, which—being a balanced compromise rather than the electrically optimal 0.1 mm—ensures good machining accuracy while maintaining reasonable processing efficiency.

(3) Experimental results demonstrate that the optimal parameter combination was achieved in Group 3-3 experiments (15 V voltage, 0.14 m/s cathode speed). Under these parameters, the machined micro-dimples exhibited an average diameter of 245.283 μm (standard deviation: 11.10 μm) and an average depth of 50.344 μm (standard deviation: 10.97 μm). These outstanding machining results demonstrate the promising potential of applying pulsating cyclic cathodes in M-ECME. By eliminating dependence on external electrolyte circulation systems, this technology achieves significantly improved cost-effectiveness and process flexibility.

## Figures and Tables

**Table 1:** Experimental content table under different parameters.

Group Number	Voltage (V)	Cathode Velocity (m/s)	Group Number	Voltage (V)	Cathode Velocity (m/s)
0-1	9	0	2-3	15	0.12
0-2	12	0	2-4	18	0.12
0-3	15	0	3-1	9	0.14
0-4	18	0	3-2	12	0.14
1-1	9	0.10	3-3	15	0.14
1-2	12	0.10	3-4	18	0.14
1-3	15	0.10	4-1	9	0.16
1-4	18	010	4-2	12	0.16
2-1	9	0.12	4-3	15	0.16
2-2	12	0.12	4-4	18	0.16

## Data Availability

The data presented in this study are available within the article.
